# Aqueous Extract and Polysaccharide of Aconiti Lateralis Radix Induce Apoptosis and G0/G1 Phase Cell Cycle Arrest by PI3K/AKT/mTOR Signaling Pathway in Mesangial Cells

**DOI:** 10.1155/2022/3664696

**Published:** 2022-04-22

**Authors:** Xingyao Li, Peng An, Yanhong Zhao, Zimo Cai, Bingyu Ye, Yafeng Wang, Wenfang Wang, Qi Gao, Liuyun Li, Tao Zhang, Xili Wu

**Affiliations:** ^1^Department of Integrated Chinese Traditional and Western Medicine, Second Affiliated Hospital of Xi'an Jiaotong University, Xi'an 710004, China; ^2^Department of Rheumatism, Xi'an Fifth Hospital, Xi'an 710082, China

## Abstract

Mesangial proliferative glomerulonephritis (MesPGN) is a common renal disease that lacks effective drug intervention. Aconiti Lateralis Radix (Fuzi), a natural Chinese medical herb, is found with significant therapeutic effects on various diseases in the clinic. However, its effects on MesPGN have not been reported. This study is aimed to discuss the therapeutic effects of the aqueous extract of Aconiti Lateralis Radix (ALR) and the polysaccharides of Aconiti Lateralis Radix (PALR) on MesPGN as well as the underlying mechanism. In this study, we, firstly, studied the anti-MesPGN mechanism of ALR and PALR. ALR and PALR inhibit the proliferation of the mesangial cells through the PI3K/AKT/mTOR pathway, induce the G0/G1 phase of block and apoptosis, inhibit the activity of Cyclin E and CDK2, increase the expression of Bax, cleaved caspase-8/caspase-8, and cleaved caspase-3/caspase-3 proteins, and effectively inhibit the growth of the mesangial cells. Overall, our data suggest that ALR and PALR may be potential candidates for MesPGN and that PALR is more effective than ALR.

## 1. Background

Mesangial proliferative glomerulonephritis (MesPGN) is a pathological type of primary glomerulonephritis mainly manifested as extracellular matrix (ECM) expansion and mesangial cell overproliferation [1]. The mesangial cells under abnormal proliferation will release inflammatory mediators, which normally lead to renal fibrosis. It will further lead to irreversible progressive glomerulus sclerosis and finally to end-stage renal diseases (ESRDs) [2]. Excessive proliferation and inflammatory reaction of the mesangial cells are pivotal in the occurrence and development of MesPGN, however, the underlying molecular mechanism is unknown, and there is no special therapeutic drug [3]. Hence, exploring the mechanism of MesPGN and finding effective intervention drugs are very urgent.

To probe into the Chinese herbal medicine prevention and cure methods of MesPGN, we should, firstly, select an appropriate inflammation model. Recently, lipopolysaccharides (LPS)-induced inflammation models are extensively applied in research on glomerulonephritis animals and at a cellular level. LPS is a phospholipid double-molecule existing on the outer walls of Gram-negative bacilli and is mainly composed of lipoid A, active polysaccharides, and O antigen. LPS is also the basic component for bacterial endotoxins to produce toxic effects, which will directly or indirectly cause excessive inflammatory reactions and immune dysfunctions *in vivo* [4]. By binding with diverse membrane receptors, LPS can initiate cellular signal conduction and NF-*κ*B/MAPK pathways and release inflammatory factors (e.g., IL-1, IL-6, TNF-*α*, and NO) to induce inflammatory reactions *in vivo* [5]. LPS can stimulate glomerular mesangial cells (GMCs) to produce TGF-*β*, TNF-*α*, IL-6, and other inflammatory factors, which will aggravate renal dysfunction through autocrine or paracrine [6].

Phosphatidylinositol 3-kinase (PI3K) is one key regulatory factor of various intracellular signaling pathways, and its activation is closely associated with cell proliferation, differentiation, apoptosis, and autophagia [7, 8]. Protein kinase B (AKT) is a downstream kinase of PI3K and is critically involved in cell death and survival [9]. The mammalian target of rapamycin (mTOR) is an important serine/threonine kinase and a downstream target of the PI3K/AKT pathway. High glucose can activate the mTORC1 pathway of diabetic rats and thereby activate the PI3K/AKT/mTOR pathway, inducing GMCs proliferation and mesangial matrix accumulation in diabetic rats [10]. Rapamycin can inhibit the high-glucose-stimulated GMCs proliferation in rats, indicating mTOR exists in GMCs and can effectively block mTOR activation and thereby inhibit the abnormal proliferation and growth of GMCs [11]. PI3K/AKT/mTOR signaling pathway plays an important role in cell survival, proliferation, and growth [12]. Research on the kidney biopsy specimens of glomerulonephritis patients shows that the apoptosis count of IgA kidney disease glomerulus cells is far larger than that of other pathological types, and MesPGN cell apoptosis is associated with albuminuria progression, indicating that glomerulus cell apoptosis is involved in glomerulus damages [13]. In addition to inhibiting apoptosis, another major role of the PI3K/AKT/mTOR pathway is that its inactivation or inhibition can lead to cell cycle arrest [14]. Cyclin-dependent kinase 2 (CDK2) and cyclin-dependent kinase inhibitor (CKI) p27 play important roles in regulating cell cycle progression or arrest [15]. The imbalance of the PI3K/AKT signaling pathway has been found in many types of kidney disease [16]. Therefore, the inhibition of the PI3K/AKT signaling pathway may be a therapeutic target for kidney diseases [17]. More reports have described that the PI3K/AKT/mTOR pathway is closely associated with the occurrence and development of MesPGN, and drugs that can inhibit its expression or lower its activity may be a new clue for the clinical treatment of MesPGN in the future [18–20].

Aconiti Lateralis Radix, a representative warm-hot drug in traditional Chinese medicine, has the efficacy of collapse rescuing and Yang restoration, fire tonification and Yang invigoration, dampness and cold dispelling, and pain-killing [21]. Modern pharmacology proves it has cardiotonic, analgesic, anesthetic, anti-inflammatory, antitumor, immune-strengthening, and kidney-protecting effects [22]. Aconiti Lateralis Radix is applied clinically to treat heart diseases, pulmonary diseases, digestive system diseases, and renal diseases. After the purification of polysaccharides, the immunomodulatory, anti-inflammatory, antidepression, antioxidation, antiageing, and antitumor pharmacological effects of Chinese medical herbs can be significantly enhanced [23–25]. The polysaccharides of Aconiti Lateralis Radix (PALR) have attracted wide attention recently because of their promyocardial, anti-inflammatory, antidepressive, antitumor, and immune-modulating effects, as well as their nontoxicity [26, 27]. Other studies and our previous research indicate that Aconiti Lateralis Radix can significantly improve some disease models, including chronic heart failure [28], acute liver failure [29], and doxorubicin-induced nephropathy diseases [30]. Hence, further research and knowledge on Aconiti Lateralis Radix are very necessary. In this study, the aqueous extract of Aconiti Lateralis Radix (ALR) and PALR were used to separately intervene human mesangial cells (HMCs). Targeting at the PI3K/AKT/mTOR pathway, we probed into the underlying mechanisms for the prevention and cure of MesPGN.

In this study, HMCs were cultured in vitro, and the effects of ALR and PALR on cell proliferation were studied by the methyl thiazolyl tetrazolium (MTT) method. The next two aspects were apoptosis and cell cycle. The cell cycle was detected by the PI flow cytometry method, and the expression of p27, CDK2, and Cyclin E was detected by western blot. Apoptosis was detected by the Annexin V-PE/7AAD method, and the expression of apoptosis-related proteins in the Bax family and Caspase family were detected by western blot. Besides, the role of the PI3K/AKT/mTOR pathway in LPS-induced cell cycle arrest and apoptosis was investigated. The study focused on the effects of ALR and PALR on proliferation, apoptosis, and the cell cycle of HMCs, and it tried to elucidate its molecular mechanism. Our results are expected to offer some clinical assistance for the treatment of MesPGN.

## 2. Methods

### 2.1. Drugs and Reagents

The aqueous extract of Aconiti Lateralis Radix (ALR) and the polysaccharides of Aconiti Lateralis Radix (PALR) were the processed products (Heishunpian) of Aconiti Lateralis Radix bought from Beijing Tongrentang Slices Ltd. Co. (No. 161201). Lipopolysaccharides (LPS, from E. Coli 055:B5) (L2880, Sigma, USA, 25 mg) were diluted by phosphate buffer solution (PBS) into a 2.5 mg/ml mother solution, which was sealed and stored in a refrigerator at −20°C. Methylprednisolone (MP) (T59405, 40 mg/ml) was bought from Pfizer Manufacturing Belgium NV.

ALR: medical slices (25 g) were soaked in 1 L of water for 30 min, then decocted for 1 h and filtered, from which the medical liquids were preserved. After that, 1 L of fresh water was added for 1 h of decoction. The liquids from the two times of decoction were combined and decocted into 250 ml. The concentration of the extractant can be calculated as 100 mg/mL by the amount of raw medicine. Next, the product was subpacked and sealed into high-pressured glass bottles, which were stored at 4°C.

PALR: medical slices (250 g) were soaked in 2.5 L of water for 30 min. They were then decocted for 1 h and filtered, from which the medical liquids were preserved. After that, 2.5 L of fresh water was added for 1 h of decoction. The liquids from the two times of decoction were combined and decocted for 5 h. Then, the supernatant was collected and added to ethanol (80% of total volume). After the mixture was placed at 4°C for 24 h, the precipitates were dissolved in water and filtered. The filtrate was added to ethanol to 80% of the total volume. The new mixture was placed at 4°C for 24 h and then centrifuged at 3000 rpm for 10 min. The precipitates were washed with acetone 3 times and then centrifuged at 3000 rpm for 10 min. The new precipitates were dissolved in water and uniformly mixed with 10% trichloroacetic acid, followed by placement at −20°C for 12 h and centrifugation at 6000 rpm for 20 min. Then, the supernatant was collected, heated in boiled water for 30 min, taken out, and cooled, followed by the addition of 400 ml of 95% ethanol and placement at 4°C for 24 h. After the liquids were fully volatilized, the polysaccharide powder was decompressed and dried at 50°C and stored at normal temperature. The concentration of the extractant can be calculated as 10 mg/mL by the amount of raw medicine.

### 2.2. High-Performance Liquid Chromatography (HPLC) Fingerprinting of the Extracts

The chemical constituents of Aconiti Lateralis Radix extracts were identified by HPLC fingerprinting analysis. Benzoylhypacoitine, benzoylmesaconine, mesaconitine, benzoylaconitine, aconitine, and hypaconitine were used as standard substances. The 100 mg/mL Aconiti Lateralis Radix extracts were filtered with a 0.22-*μ*m microporous membrane. Samples were passed through an Eclipse C_18_ column (4.6 mm × 250 mm, 5 *μ*m) at a flow rate of 0.8 mL/min with the mobile phases of 0.2% ammonia (*A*) and acetonitrile (B). Column separation for alkaloids was performed by a gradient elution program: 0–15 min, 24–29% B; 15–50 min, 29%–50% B; 50–75 min, 50–78% B. The column temperature was 35°C.

### 2.3. Cell Culture and Treatment

The fifth-generation human mesangial cells (HMCs) purchased from Guangzhou Jennio Biotech Co., Ltd. (China) were cultured in a cell incubator (37°C, 5% CO_2_) added with Dulbecco's minimum essential medium (DMEM, HyClone, Carlsbad, CA, USA), which was supplemented with 10% fetal bovine serum (FBS, Gibco, Grand Island, NY), 100 U/ml penicillin, and 100 mg/ml streptomycin (Gibco, Grand Island, NY).

### 2.4. Cell Proliferation

The HMCs at the logarithmic phase were inoculated into 96-well culture plates (1 × 10^5^ cells/well). Five groups were set (all *n* = 6): a control group (Ctrl), an LPS model group (LPS, 100 *μ*g/ml), an Aconiti Lateralis Radix aqueous extract group (ALR, 10 mg/ml), an Aconiti Lateralis Radix polysaccharide group (PALR, 0.1 mg/ml), and a positive control methylprednisolone group (MP, 0.3 mg/ml). After the cells fully adhered to the walls, the cells were periodically synchronized for 24 h in DMEM containing 0.5% (v/v) FBS. The control group was added with 100 *μ*l of DMEM, while other groups were added with 100 *μ*l of 100 *μ*g/ml LPS. After 12 h of stimulation, the three drug groups were added with 100 *μ*l of ALR, PALR, and MP, respectively, but the control group and the LPS group were both added with 100 *μ*l of DMEM, followed by 24 h of intervention. Each group was set with six 200-*μ*l compound wells, and the marginal wells were filled with PBS. After 24 h, the HMCs in each group were counted using a Coulter counter (Beckman, Brea, USA) and observed under a microscope (Nikon, Tokyo, Japan). Each well was added with 20 *μ*L of 5 mg/mL MTT (Sigma) and cultured in a 5% CO_2_ incubator at 37°C and in dark for 4 h. Then, the supernatant was sucked out, and each well was added with 150 *μ*L of dimethyl sulfoxide (DMSO, Shanghai Hushi, China) while shaking at room temperature for 10 min, which ensured complete crystallization and dissolution. Then, the absorbance (*A*) at 570 nm was measured by an enzyme labeling meter (Thermo, USA), and the cell survival rate was calculated.

### 2.5. Cell Cycle Analysis

The HMCs were washed with PBS, digested, and made into a cell suspension using DMEM containing 0.5% FBS. After cell counting, the cells were inoculated into 6-well plates (3 × 10^5^/2 mL per well). Then, after full-medium cultivation for 24 h, the medium was discarded and changed with a medium containing only 0.5% FBS for 24 h, which ensured cell synchronization. The media from the control group, test groups, and positive control group (each 2 mL) were added separately. After further routine cultivation for another 24 h, cells were collected by centrifugation and washed with PBS two times. After the removal of supernates, 5 mL of precooled 70% ethanol was added to beat the cells into unicellular suspensions, which were fixed and stored at 4°C. After washing with PBS two times and centrifugation at 1000 rpm/min for 10 min, the cells were stained with propidium iodide (PI, final concentration = 20 *μ*g/mL) and RNaseA (final concentration = 50 *μ*g/mL) (both Becton Dickinson, SanJose, USA) at 37°C and in dark for 30 min. Then within 30 min after the cells were uniformly mixed and filtered through 200-mesh nylon webs, DNA and cell cycle distribution were detected on a flow cytometer (BD Co. Ltd., USA). The cell cycle was analyzed on ModFIT 5.0.

### 2.6. Cell Apoptosis Analysis

The HMCs were washed with PBS, digested, and made into a cell suspension using DMEM containing 0.5% FBS. After cell counting, the cells were inoculated into 6-well plates (3 × 10^5^/2 mL per well). Then, after full-medium cultivation for 24 h, the medium was discarded and changed with a medium containing only 0.5% FBS for 24 h, which ensured cell synchronization. The media from the control group, test groups, and positive control group (each 2 mL) were added separately. After further routine cultivation for another 24 h, cells were collected by centrifugation and washed with PBS two times. Cells (1 × 10^6^ cells from each group) were collected using centrifuge tubes. The cells were resuspended in 500 *μ*L of buffer, and 5 *μ*L of AnnexinV-PE or 7AAD (Becton Dickinson, SanJose, USA) was added before incubation at room temperature and in dark for 30 min, followed by detection by flow cytometry. Data were analyzed on relevant software.

### 2.7. Immunofluorescence

The HMCs were washed with PBS, digested, and made into a cell suspension using DMEM containing 0.5% FBS. After cell counting, the cells were inoculated into 6-well plates (3 × 10^5^/2 mL per well). Then, after full-medium cultivation for 24 h, the medium was discarded and changed with a medium containing only 0.5% FBS for 24 h, which ensured cell synchronization. The media from the control group, test groups, and positive control group (each 2 mL) were added separately. After routine cultivation for another 24 h, the cells were washed by PBS three times, fixed with 4% paraformaldehyde for 15 min, and washed with PBS three more times. Then, the cells were sealed by dripping 5% normal goat serum for 30 min, directly dripped with rabbit VEGF monoclonal antibody (1 : 100), and incubated at normal temperature for 90 min. After washing with PBS three times, FITC-labeled goat antirabbit fluorescent secondary antibody (1 : 100) was incubated for 60 min at room temperature and in dark. After washing with PBS three times, DAPI-labeled (Wuhan Guge Biological Technology Co., Ltd.) cells were added for 10 min of incubation at room temperature and in dark. After washing with PBS three times, the cells were coated with sealing tablets and observed under a laser confocal microscope (Lecia, Germany) at 400 magnification.

### 2.8. Western Blot

Total proteins were extracted and added with a RIPA lysis buffer (Thermo, US). Protein contents were measured using a bicinchoninic acid (BCA) protein kit (Beyotime, Shanghai, China). Then, 10% sodium dodecyl sulfate-polyacrylamide gel electrophoresis (SDS-PAGE) gels were prepared for electrophoretic protein separation. The proteins were transferred to polyvinylidene fluoride (PVDF) membranes and sealed in 5% skim milk for 1 h. After washing with PBS three times, the cells were added with AKT (#4691), mTOR (#2983), p-AKT (#4060), p-mTOR (#5536), p27 (#3686), CDK2 (#2546), CyclinE (#4129), Bax (#5023), Cleaved Caspase-8 (#8592), Caspase-8 (#4790), Cleaved Caspase-3 (#9664), Caspase-3 (#9662) (all from Cell Signaling Technology, Danvers, MA, USA), and *β*-actin (F170031, Abways, Shanghai, China), sealed and placed overnight at 4°C. Then, the corresponding HRP-labeled goat antirabbit IgG secondary antibody (P48010, Yeasen, Shanghai, China) was added for 1 h of incubation. Finally, the membranes were displayed by a chemiluminescence agent (Bio-Rad, USA). Western blot data were analyzed on ImageJ.

### 2.9. Statistical Analysis

All data were expressed as ($\bar{x}$ ± *S*). Intergroup differences were compared by one-way analysis of variance (ANOVA) on SPSS 23.0 (Chicago, IL, USA). Data were statistically plotted on Prism7.0. The significant level was *P* < 0.05.

## 3. Results

### 3.1. HPLC Fingerprinting of Aconiti Lateralis Radix

**Figure 1:**
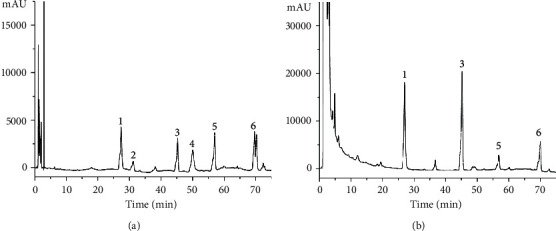
HPLC fingerprint chromatogram of Aconiti Lateralis Radix extracts. (a) HPLC fingerprint chromatograms of the extracts of the reference standards. (b) Aconiti Lateralis Radix. In the chromatograms, (1) benzoylhypacoitine (PubChem CID:3047329); (2) benzoylmesaconine (PubChem CID:13343340); (3) mesaconitine (PubChem CID:416228); (4) benzoyl aconitine (PubChem CID:1221732); (5) aconitine (PubChem CID:245005); (6) hypaconitine (PubChem CID:441737).

As is known to all, Aconiti Lateralis Radix is a kind of traditional Chinese medicine with both toxicity and efficacy. Rational use can bring therapeutic effects into play, while improper use may threaten safety and health. The decocted Aconiti Lateralis Radix helps to reduce the toxicity of the drug and might exert a large maximal therapeutic efficacy with minimal adverse effect [31]. Therefore, we conducted HPLC detection on the decocted Aconiti Lateralis Radix. The HPLC fingerprint chromatogram of Aconiti Lateralis Radix extracts of the reference standards is shown in Figure 1(a). Benzoylhypacoitine, mesaconitine, aconitine, and hypaconitine were well-identified in Aconiti Lateralis Radix extracts by comparing both retention times and UV spectra (Figure 1(b)). The results showed that the relative contents of benzoylaconitine and mesaconitine were the highest in Aconiti Lateralis Radix, and the contents of diester alkaloids, such as aconitine and hypaconitine were very low, indicating that after decocting Aconiti Lateralis Radix, the composition of highly toxic diester C_19_ diterpenoid alkaloids decreased, and the content of less toxic monoester alkaloids increased, which was consistent with the reported study [32, 33]. Research demonstrated that compounds mesaconitine and benzoylhypacoitine might be the principal active components of Aconiti Lateralis Radix for the main activities of energy metabolism [34], which just confirmed our results. Therefore, it can be shown that the use of Aconiti Lateralis Radix reduces toxicity and increases the efficacy function. In this study, the ALR was extracted from Aconiti Lateralis Radix, while PALR was a monomer of Aconiti Lateralis Radix.

### 3.2. ALR and PALR Both Inhibited and LPS Promoted Growth of HMCs

**Figure 2:**
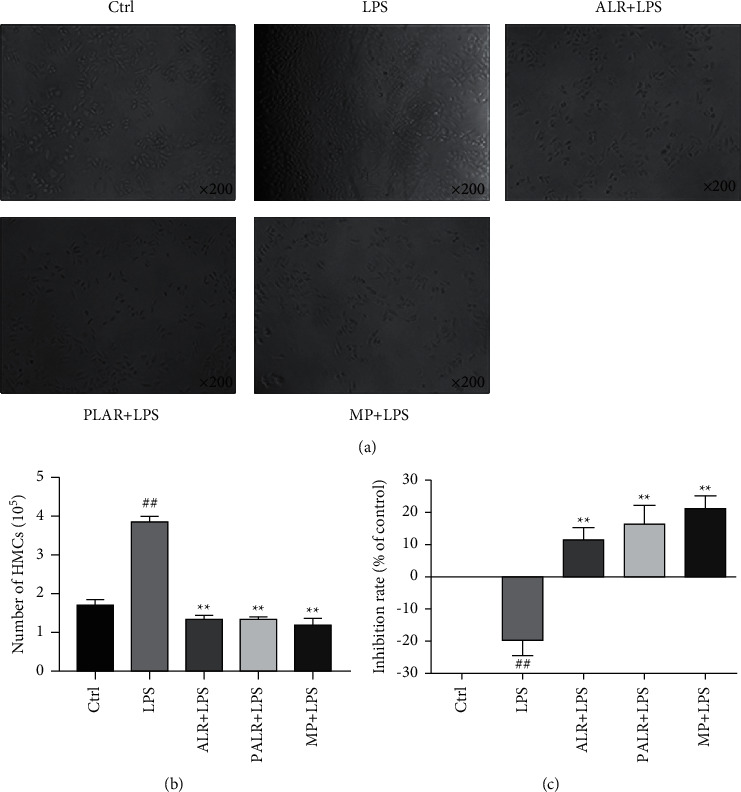
ALR and PALR inhibited the proliferation of HMCs. (a) The morphology of ALR (10 mg/ml), PALR (0.1 mg/ml), and LPS-treated (100 *μ*g/ml) HMCs for 24 h. The control group was added with 100 *μ*l of DMEM, while other groups were each added with 100 *μ*l of 100  *μ*g/ml LPS. After 12 h of stimulation, the three drug groups were added with 100 *μ*l of ALR, PALR, and MP, respectively, however, the control group and the LPS group were both added to 100 *μ*l of DMEM, followed by 24 h of intervention. Images were taken using a microscope (200 × magnification). (b) The number of the HMCs counted using a Coulter Counter. All values are expressed in mean ± SD (*n* = 3; ^##^*P* < 0.01 versus control group; ^*∗∗*^*P* < 0.01 versus LPS group). (c) The inhibition rate of HMCs in each group. All values are expressed in mean ± SD (*n* = 6; ^##^*P* < 0.01 versus control group; ^*∗∗*^*P* < 0.01 versus LPS group).

To evaluate the effects of ALR and PALR on HMCs, the morphological changes of the HMCs after 24 h of culture were observed microscopically (Figure 2(a)), and the HMCs were statistically counted (Figure 2(b)). Results showed the HMCs were concentrated after induction by LPS (100 *μ*g/ml), and ALR (10 mg/ml) and PALR (0.1 mg/ml) both decreased cell density. The HMCs under induction by LPS grew rapidly, and ALR and PALR both inhibited the proliferation of HMCs. To validate the protective effect on HMCs, the HMCs were incubated with 100 *μ*g/ml LPS and the optimal concentration of ALR or PALR for 24 h. MTT experiments showed LPS significantly improved the activity of HMCs (*P* < 0.01), and ALR, PALR, and MP significantly inhibited the proliferation of LPS-induced HMCs (*P* < 0.01) (Figure 2(c)).

### 3.3. ALR and PALR Inhibited Proliferation and Affected Cycle of HMCs

**Figure 3:**
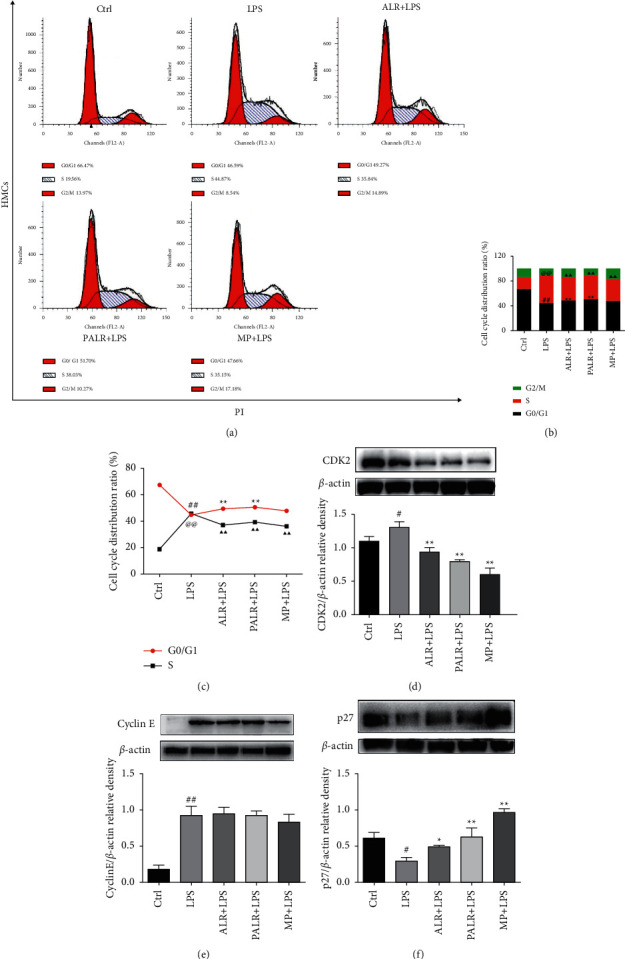
ALR and PALR affect the cell cycle of HMCs. HMCs were stained with PI for flow cytometry analysis. (a) Representative images of cell cycle. (b-c) The rate of G0/G1 phage, S phage, and M phage (%). (d-f) The protein expressions of CKD2, Cyclin E, and p27 were analyzed using western blot. The statistical data of the proteins were analyzed with Image J 1.8.0 software. Data were expressed as mean ± SD (*n* = 3; ^#^*P* < 0.05,^##^*P* < 0.01 or ^@@^*P* < 0.01 versus control group, ^*∗*^*P* < 0.05, ^*∗∗*^*P* < 0.01 or ^▲▲^*P* < 0.01versus LPS group).

The effects of ALR and PALR on the proliferation of HMCs were further evaluated by cell cycle analysis. As shown in Figures 3(a)–3(c), LPS decreased the proportion of G0/G1-phase cells but increased the proportion of the S-phase cells, indicating LPS promoted cell cycle progression (*P* < 0.01). In the ALR and PALR groups, the proportion of G1 phase cells rose, however, the proportion of the S-phase cells decreased (*P* < 0.01), suggesting ALR and PALR both can inhibit the G1-S phase transformation of LPS-induced cells and block the G0/G1 phase cells, thereby blocking cell cycle progression.

To clarify whether ALR and PALR can inhibit the G1 phase of HMCs, we detected the Cyclin E, CDK2, and p27 expressions by Western blot. Cyclin E/CDK2, as key genes of cell cycle regulation, are critical kinase compounds of cells entering from phase G1 to phase S, and they control the G1-S transformation in the cell cycle [35]. p27 can severely inhibit Cyclin E and CDK2, blocking the cells at phase G1 and inhibiting cell proliferation [36]. Results show that LPS negatively regulates p27 and induces the expressions of Cyclin E and CDK2 (*P* < 0.05), but ALR and PALR upregulate p27 expression (*P* < 0.05) and downregulate CDK2 expression (*P* < 0.01) (Figures 3(d)–3(f)). LPS promotes the proliferation of HMCs, while ALR and PALR inhibit G1-S transformation and block HMCs at phases G0/G1.

### 3.4. Effects of ALR and PALR on Cell Apoptosis and Related Proteins

**Figure 4:**
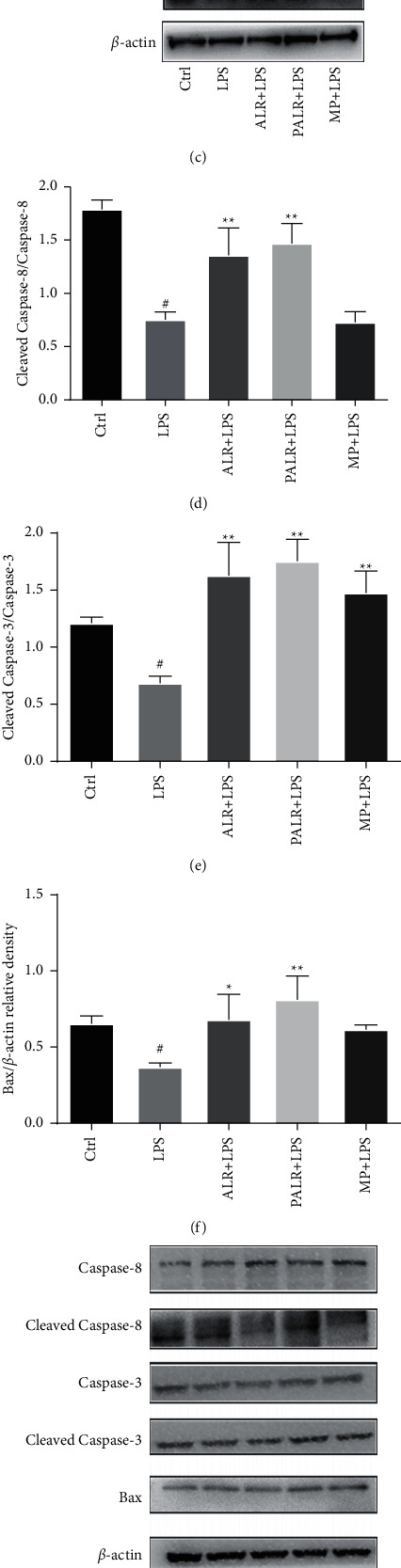
The induction of cell apoptosis arrest by ALR and PALR in HMCs. (a) Representative images of cell apoptosis. Flow cytometry analysis revealed that ALR (10 mg/ml) and PALR (0.1 mg/ml) induced the apoptosis of HMCs, as determined by AnnexinV-PE/7AAD. (b) The apoptotic rate of HMCs in each group. All data are depicted as mean ± SD (*n* = 3; ^##^*P* < 0.01 versus control group, ^*∗∗*^*P* < 0.01 versus LPS group). (c–j) The protein expressions of Bax, cleaved caspase-8/caspase-8, and cleaved caspase-3/caspase-3 were analyzed using Western blot. HMCs in (c–f) were induced by LPS. However, there was non-LPS induction in (g–j). All data are depicted as mean ± SD (*n* = 3; ^#^*P* < 0.05 or ^##^*P* < 0.01 versus control group, ^*∗*^*P* < 0.05 or ^*∗∗*^*P* < 0.01 versus LPS group).

To validate whether ALR and PALR can induce apoptosis of HMCs, we detected the apoptosis of HMCs at 24 h after intervention by ALR (10 mg/ml) and PALR (0.1 mg/ml) using AnnexinV-PE/7AAD double staining. Results showed that the apoptosis rate significantly declined in the LPS group (*P* < 0.01) but significantly rose in the ALR and PALR groups (*P* < 0.01), suggesting ALR and PALR can promote the apoptosis of HMCs under induction by LPS (Figures 4(a) and 4(b)). Bax, cleaved caspase-8/caspase-8, cleaved caspase-3/caspase-3 expressions were further detected by Western blot. As shown in Figures 4(c)–4(f), ALR and PALR can upregulate Bax expression (*P* < 0.05), cleaved caspase-8/caspase-8 (*P* < 0.01), and cleaved caspase-3/caspase-3 expression (*P* < 0.01), indicating ALR and PALR both can promote the apoptosis of HMCs under the inflammatory status, and apoptosis may be realized jointly by endogenous and exogenous pathways. To explore whether ALR and PALR can promote the normal apoptosis of HMCs, we detected apoptin expression in normal HMCs. Results showed Bax, cleaved caspase-8/caspase-8, and cleaved caspase-3/caspase-3 expressions in the LPS group were not significantly different from the control group (*P* > 0.05). Bax, cleaved caspase-8/caspase-8, and cleaved caspase-3/caspase-3 expressions were not significantly different in the ALR, PALR, and MP groups compared with the LPS group (*P* > 0.05). Hence, it is clear that ALR and PALR within certain concentrations do not affect the physiological apoptosis of HMCs, It can be seen that ALR and PALR do not affect the physiological apoptosis of HMCs at a certain concentration, which once again proves that the ALR and PALR used in our study are safe (Figures 4(g)–4(j)).

### 3.5. Effects of ALR and PALR on the PI3K/AKT/mTOR Pathway

**Figure 5:**
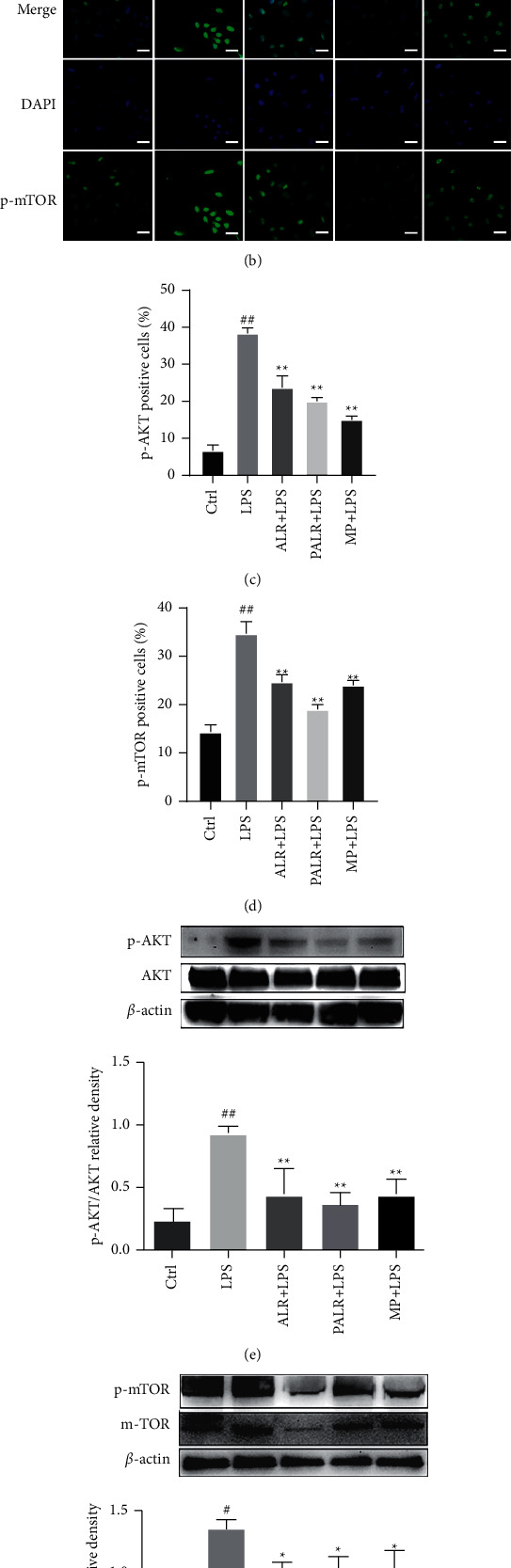
ALR and PALR mediate the PI3K/AKT/mTOR signaling pathway in HMCs. (a-b) Representative p-AKT and p-mTOR results observed using a light microscope (400 × magnification) (scale bar = 20 *μ*m). (c-d) The quantification results of p-AKT positive cells and p-mTOR positive cells (%). Data were expressed as mean ± SD (*n* = 3; ^##^*P* < 0.01 versus control group, ^*∗∗*^*P* < 0.01 versus LPS group). (e-f) The protein expressions of p-AKT and p-mTOR were analyzed using Western blot. Data were expressed as mean ± SD (*n* = 3; ^#^*P* < 0.05 or ^##^*P* < 0.01 versus control group, ^*∗*^*P* < 0.05 or ^*∗∗*^*P* < 0.01 versus LPS group).

To clarify the molecular mechanism of how ALR and PALR inhibit the proliferation of LPS-induced HMCs, laser confocal microscopy demonstrated after the intervention of LPS-induced (100 *μ*g/ml) HMCs by ALR (10 mg/ml) and PALR (0.1 mg/ml), the p-AKT, and p-mTOR were significantly weakened in fluorescence intensity and expression, and the cell morphology gradually changed from a normal spindle, or star shape, to the ellipse shape (Figures 5(a)–5(d)). It was indicated that ALR and PALR can inhibit p-AKT and p-mTOR to some extent. Western blot showed that p-AKT/AKT and p-mTOR/mTOR expressions in HMCs declined (*P* < 0.05), which is consistent with the results of laser confocal microscopy (Figures 5(e) and 5(f)). It is suggested that ALR and PALR may attenuate the injuries to HMCs by downregulating the PI3K/AKT/mTOR pathway.

## 4. Discussion

The proliferation of GMCs will directly progress to MesPGN, which is a common glomerulus disease in clinics. Its basic symptoms include albuminuria, hypertension, and edema, accompanied by different degrees of renal dysfunction. Without prompt control, MesPGN is progressive and will finally develop to ESRD [37]. Interventions that inhibit the proliferation and ECM deposition of inflammatory GMCs are important for delaying the progression of MesPGN. So far, the modern medical treatment of MesPGN is still dominated by glucocorticoids and other immunosuppressants, however, despite the significant therapeutic effects, such treatment is still limited by high recurrence rate and severe side effects. For this reason, from the aspect of traditional Chinese medicine, developing effective Chinese medical herbs with low toxicity and side effects will bring new hope for the treatment and prognosis improvement of MesPGN and for delaying the occurrence of renal function failure. Aconiti Lateralis Radix is the daughter root processed product of Aconitum carmichaelii Debeaux, and its unknown chemical components mainly include alkaloids, polysaccharides, fatty acids, and phosphatidic calcium of Aconiti Lateralis Radix, *β*-sitosterol, flavonoids, saponins, and fatty acids [22]. Aconiti Lateralis Radix tastes pungent and sweet, and it is extremely hot in nature. It has anti-inflammatory, analgesic, antiepileptic, and vascular-protective effects [38]. Aqueous extract and polysaccharides of Aconiti Lateralis Radix have various pharmacological activities, however, the therapeutic mechanism of MesPGN is still unknown. For this reason, we explored the aqueous extract and polysaccharides of Aconiti Lateralis Radix at the cell molecular level.

In this study, HMCs were irritated by 100 *μ*g/ml LPS to preliminarily investigate the changes of HMCs at the early excessive inflammation stage. After stimulation by LPS, HMCs bloomed and significantly increased in cell count. MTT experiments showed that the OD of the LPS group was significantly higher than that of the control group, and the inhibition ratio was negative, indicating LPS can obviously promote the proliferation of HMCs and that the inflammation model was successfully built.

In the exogenous pathways of apoptosis, death receptors and ligands bind together and migrate intracellularly, and then the death domain (DD) inside death receptors recruits several molecules of pro-Caspase-8 to form death-inducing signaling complex (DISC). After that, the DD is self-cut and activated to form caspase-8, and it further activates downstream caspase-3 to induce apoptosis [39].

In the endogenous pathways, Bcl-2 proteins are pivotal in cell apoptosis. Bax, the earliest-discovered proapoptotic protein, is mainly distributed in the cytoplasms, and after irritation by apoptotic signals, it will be transferred to mitochondria, releasing Cyt-C. Cyt-C released to the cytoplasts will bind with Apaf-1 to form polymers and promote the binding between pro-Caspase-9 and apoptosome, activating caspase-9 and downstream caspase-3 and caspase-7, which will finally lead to cell apoptosis [40]. The caspase protein family is an important member of the cell regulatory genes, which are involved in the initiation of apoptosis and the regulation of the whole process, in which caspase-3 is a key protease activated by various apoptotic stimuli [41]. In our study, Western blot showed that ALR and PALR could upregulate the cleaved caspase-8/caspase-8, cleaved caspase-3/caspase-3, and Bax protein expressions, suggesting that apoptosis may be caused jointly by endogenous and exogenous pathways. The effect of PALR was better than that of ALR.

The PI3K/AKT/mTOR signaling pathway plays an important role in cell survival, proliferation, and growth. In addition to inhibiting apoptosis, another major role of the PI3K/AKT/mTOR pathway is that its inactivation or inhibition can lead to cell cycle arrest [14]. Therefore, it has been reported that blocking the PI3K/AKT signaling pathway can inhibit LPS-induced abnormal proliferation and growth of HMCs, and Celastrol (CLT) can inhibit the proliferation of MesPGN model rats and HBZY-1 cell, and cell arrest was induced in G0/G1 phase, which showed a significant increase in the number of cells in the G0/G1 phase and a significant decrease in the number of cells in the S phase, which was consistent with our results [42]. In this study, we investigated whether ALR and PALR affected the PI3K/AKT/mTOR signaling pathway. Western blot results showed ALR and PALR could reduce the expression of p-AKT/AKT and p-mTOR/mTOR, indicating that ALR and PALR inhibited the PI3K/AKT/mTOR signaling pathway. In conclusion, ALR and PALR inhibit the growth of LPS-induced HMCs by blocking the cell cycle and increasing apoptosis through the PI3K/AKT/mTOR signaling pathway.

## 5. Conclusions

In summary, ALR and PALR can induce mesangial cells apoptosis and G0/G1 cell cycle arrest through the PI3K/AKT/mTOR signaling pathway, which is a potential mechanism for effective prevention of MesPGN, and the effect of PALR is better than that of ALR. However, additional evidence from future studies involving animals is required to confirm the role of ALR and PALR in MesPGN.

## Data Availability

The datasets used during this study are available from the corresponding author upon reasonable request.
